# Endocytic Trafficking of Membrane-Bound Cargo: A Flotillin Point of View

**DOI:** 10.3390/membranes4030356

**Published:** 2014-07-11

**Authors:** Melanie Meister, Ritva Tikkanen

**Affiliations:** Institute of Biochemistry, Medical Faculty, University of Giessen, Friedrichstrasse 24, 35392 Giessen, Germany; E-Mail: melanie.meister@biochemie.med.uni-giessen.de

**Keywords:** flotillin, endocytosis, lipid microdomains, clathrin independent endocytosis, dynamin, endosomal sorting, recycling, exosomes

## Abstract

The ubiquitous and highly conserved flotillin proteins, flotillin-1 and flotillin-2, have been shown to be involved in various cellular processes such as cell adhesion, signal transduction through receptor tyrosine kinases as well as in cellular trafficking pathways. Due to the fact that flotillins are acylated and form hetero-oligomers, they constitutively associate with cholesterol-enriched lipid microdomains. In recent years, such microdomains have been appreciated as platforms that participate in endocytosis and other cellular trafficking steps. This review summarizes the current findings on the role of flotillins in membrane-bound cargo endocytosis and endosomal trafficking events. We will discuss the proposed function of flotillins in endocytosis in the light of recent findings that point towards a role for flotillins in a step that precedes the actual endocytic uptake of cargo molecules. Recent findings have also revealed that flotillins may be important for endosomal sorting and recycling of specific cargo molecules. In addition to these aspects, the cellular trafficking pathway of flotillins themselves as potential cargo in the context of growth factor signaling will be discussed.

## 1. Lipid Microdomains and Endocytosis

Initially, lipid microdomains were described in the early 1990s as membrane structures that are insoluble in cold non-ionic detergents such as Triton X-100 and thus float in low density fractions [1]. Ever since Simons and Ikonen proposed the principle of lipid rafts in 1997 [2], it has been refined over the years. Nowadays, such lipid microdomains are considered as specific nanoscale assemblies enriched in cholesterol and sphingolipids that constitute a liquid-ordered phase in cellular membranes. These microdomains are dynamic and can coalesce to serve as signaling platforms or to function in membrane trafficking [3].

Certain modifications and properties of proteins increase their propensity to associate with lipid microdomains. For example, the association of glycosylphosphatidyl-inositol (GPI) anchored proteins with rafts is mediated by their glycolipid anchor [4,5]. Multiple acylation has been shown to enhance the affinity of proteins for the liquid-ordered membrane phase. For example, tyrosine kinases of the Src family are doubly acylated and thus associate with rafts [5,6,7,8]. Palmitoylation, which is a reversible process that takes place in Cys residues, was suggested to serve as regulatory means to recruit or exclude proteins from lipid microdomains [9]. However, single palmitoylation alone is not sufficient to recruit proteins into rafts, as evidenced by the transferrin receptor which can be palmitoylated but is constitutively localized outside of rafts [9]. Apart from GPI anchors and acylation, oligomerization of proteins enhances their affinity for rafts and can also serve to stabilize the respective microdomain in a scaffolding manner [9,10,11,12].

Endocytosis can be roughly classified in two categories: clathrin mediated endocytosis (CME) and clathrin independent endocytosis (CIE). For recent reviews, the reader is referred to [13,14,15,16]. In contrast to the detailed mechanistic insights that are available for CME and its structural component clathrin, CIE is far less understood. So far, it appears that a major hallmark of CIE is that even uncoated membrane pits can be invaginated and internalized into the cell. While CME depends on dynamin for vesicle scission, both dynamin dependent and independent CIE pathways have been described. The fission of caveolae, invaginated structures in the plasma membrane that are decorated with caveolins and cavins [17], depends on dynamin [18,19]. On the other hand, flotillin mediated endocytosis of some cargo molecules was suggested to be dynamin independent [20,21], whereas the growth factor induced internalization of flotillins clearly depends on dynamin [22]. In this review, we will only shortly summarize the suggested role of flotillins in CIE. For a more comprehensive review on flotillins in the endocytosis of specific cargo molecules, please refer to a recent review by Otto and Nichols [23]. The purpose of the present review is to critically discuss recent findings that suggest that in the case of some cargo molecules, flotillins might not actively participate in endocytosis but rather in a step preceding the endocytic uptake that may even take place by means of CME. In addition, we will provide insights into the emerging role of flotillins in cargo sorting within endosomes.

## 2. The Flotillin Protein Family

Flotillin-1/reggie-2 and flotillin-2/reggie-1 constitutively associate with specific membrane microdomains by acylation (a single palmitate in flotillin-1, a myristate and three palmitates in flotillin-2) [11,24,25], oligomerization [11,12,21,26] and cholesterol binding ([27]; our unpublished data). Upon the discovery by Bickel *et al.,* flotillins were implicated to exert a functional role in membrane trafficking processes [28]. Originally, it was proposed that flotillins associate with caveolae [28,29], but later findings clearly have shown that flotillins participate in the formation of specific non-caveolar microdomains [20,30]. Furthermore, our unpublished results from flotillin-2 knockout mice do not reveal any significant changes in caveolin protein expression. Nowadays, flotillins are commonly used as marker proteins for non-caveolar rafts. Their ability to float in low density fractions of Triton X-100 insoluble membrane preparations coined their name as flotillins and indicated their association with rafts [28].

Structurally, flotillins are composed of two domains, the function of which has not been clarified in detail. The N-terminal SPFH (stomatin/prohibitin/flotillin/HflK/C) domain contains the sites for acylation [11,24,25,27,31], whereas the so-called flotillin domain in the C-terminus mediates the oligomerization and contains Ala-Glu repeats and phosphorylatable tyrosines that are important for flotillin function [11,12,26,32,33,34]. Both flotillins are ubiquitously expressed, conserved among species and homologous to each other [35,36], although they appear to be functionally distinct. However, the expression of one flotillin depends on that of the other one, and depletion or deletion of one flotillin also reduces the stability of the other. However, flotillin-1 appears to be more dependent on flotillin-2 than vice versa [26,37,38]. Functionally, flotillins have been implicated in several cellular processes, such as cellular migration and adhesion, signaling by receptor tyrosine kinases and mitogen activated protein kinases (MAPK) as well as membrane trafficking. For detailed information on the role of flotillins in signal transduction and putative roles in cancer, we would like to refer the reader to our recent review articles [34,39,40].

Flotillins display a dynamic cellular localization that considerably varies between different cell types [21,31]. Under growth conditions, flotillins predominantly localize to the plasma membrane and endosomal structures, *i.e.*, late endosomes, recycling endosomes and exosomes [12,27,31,41,42,43,44,45,46]. However, under growth factor deprivation, flotillins relocate to the plasma membrane by means of recycling from intracellular compartments. Upon stimulation with epidermal growth factor (EGF), Src family kinases phosphorylate flotillins at several tyrosine residues, and flotillin oligomers increase in size and translocate to late endosomes [12,32,33]. Furthermore, flotillins actively participate in signaling pathways, e.g., receptor tyrosine kinase signaling and MAPK signaling [37,38,39,40,47].

## 3. Discovery of the Putative Flotillin Dependent Endocytosis Pathway

To date, several cargo molecules, such as the GPI-anchored protein CD59, cholera toxin B subunit (CTxB), cationic molecules and polyplexes, proteoglycans and proteoglycan bound ligands, as well as the Niemann-Pick C1-like 1 protein (NPC1L1) [20,21,48,49,50] have been suggested to utilize an internalization pathway that depends on flotillin-1 (Table 1). The initial idea that flotillins would establish their own CIE pathway, was suggested by Glebov *et al.*, who found increasing amounts of flotillin-1 in early endocytic vesicles after fluid-phase uptake of magnetic nanoparticles (ferrofluid) [20]. However, they did not observe a colocalization of flotillin-1 with transferrin (Tfn), a classical cargo for CME, or with clathrin in these early endocytic vesicles. Due to these findings, together with the observation that flotillin-1 colocalizes in HeLa and COS-7 cells with the GPI-anchored protein CD59 and the ganglioside GM1, Glebov *et al.* reasoned that flotillins participate in an internalization pathway that is different from CME. This was further supported by the findings demonstrating that upon expression of a dominant negative version of AP180, a molecule required for the formation of clathrin coated pits (CCPs) [51], ectopically expressed flotillin-1-GFP still colocalized with CTxB in endocytic vesicles, and depletion of flotillin-1 partially inhibited the uptake of an antibody directed towards CD59 [20,52]. However, CTxB, which binds to its receptor GM1, is somewhat controversial as a raft marker, since CTxB/GM1 have been found to be internalized not only by CIE, but also via CCPs and thus CME [53,54]. Upon immunolabeling of ultra-thin cryosections, vesicles positive for flotillin-1-GFP and CTxB were detected. However, according to the authors, only 15% of the total flotillin-1-GFP was found in these vesicles, and neither CTxB nor CD59 were significantly enriched in flotillin-1-GFP positive vesicles and invaginations at the plasma membrane. Live imaging with total internal reflection of fluorescence (TIRF) showed a very dynamic behavior of flotillin-1-GFP at the plasma membrane, with vesicles that disappeared towards the cellular interior. It was observed that flotillin-1-GFP positive vesicles and microdomains at the plasma membrane are very dynamic and move with a high mean velocity as compared to CCVs [20,21]. The dynamic movement of flotillins at the plasma membrane is in line with the fluctuating and varying lifetime of lipid microdomains [55,56]. However, flotillin-1-GFP containing vesicles bud into the cell at a frequency that is less than one third of that of CCPs [20]. Pursuing the idea that flotillins would define a CIE pathway, Frick and colleagues proposed that flotillins might serve as structural components for this pathway [21]. They observed that ectopic expression of flotillin-1-GFP and flotillin-2-GFP induces their coassembly to specific flotillin microdomains which induce membrane curvature and thus generate membrane buds that in turn bud towards the cellular interior. They suggested that the highly dynamic flotillin microdomains become static just prior to their internalization [21], which might be caused by coalescence of flotillin oligomers into larger oligomeric structures, as we have shown to occur upon EGF stimulation of the cells [12]. Since the study of Frick *et al.* was based on overexpression of GFP-tagged flotillins, which do not necessarily fully resemble the endogenous proteins in terms of their trafficking and oligomerization, the suggested capability of flotillins to induce membrane buds needs to be dissected in further studies. However, this study elegantly shows that flotillins assemble into microdomains, a property which is based on their propensity to form oligomers, as has later also been observed by us and others [12,21,26].

**Table 1:** Overview of the literature on flotillins in cellular sorting and endocytosis.

Sorting Process	References
Flotillin assisted endocytosis	[37,41,50,57,58]
Polarized sorting	[59,60,61,62,63,64,65]
Exosomes	[27,44,46,66]
Endosomal sorting	[42,67,68,69]
Flotillin oligomerization	[11,12,26,32]
Flotillin dependent endocytosis	[20,21]

## 4. Flotillin Dependent Cargo Trafficking and Sorting: Beyond Endocytosis

### 4.1. Flotillins in Sorting Events within Endosomes

In recent years, a number of publications have indicated a role for flotillins in endosomal sorting processes and in the formation of exosomal vesicles in endosomes. Generation and release of exosomes frequently occurs within multivesicular bodies (MVBs), which then fuse with the plasma membrane and thereby release their intraluminal vesicles (ILV) as exosomes to the extracellular space [70,71]. Several publications have suggested a role for lipid microdomains and flotillins in exosome generation [27,44,46,72]. The tetraspanin CD63 and Alix are commonly considered as proteins enriched in exosomes and can thus be used as markers to study exosome release [66]. Strauss and colleagues found that cholesterol treatment of oligodendrocytes resulted in an increased exosome release and these exosomes were enriched in flotillin-2, Alix and EGFP-CD63 [27]. In contrast, Baietti *et al.* found that Alix and CD63, together with syntenin, reside in flotillin-negative exosomes released from MCF-7 cells [73]. According to Phuyal and colleagues, depletion of neither flotillin-1 nor flotillin-2 influenced the number of exosomes released, whereas the sorting of caveolin-1 and annexin A2 to exosomes was impaired [46]. Thus, flotillins might participate in the sorting of specific proteins towards ILVs that are destined to generate exosomes that do not contain Alix and CD63. However, detailed mechanistic insights into how flotillins sort cargo for exosomal release is still missing.

Other studies have implicated a role for flotillins in cargo recycling. Saslowsky *et al.* observed that flotillins participate in the sorting of the cholera toxin-GM1 complex from endosomes via the TGN to the ER in zebrafish [67]. Interestingly, depletion of both flotillins rendered the fish resistant to intoxication with cholera toxin. Similar results were observed in mammalian COS-1 cells, in which cholera toxin requires flotillins to exert its cytotoxic effects. Interestingly, the averted toxicity of cholera toxin in flotillin depleted cells was shown not to be due to a reduced binding of cholera toxin to the plasma membrane GM1 or a defect in endocytosis, but rather due to a defect in the transport of cholera toxin from the plasma membrane to the ER [67]. Since neither the binding of cholera toxin to GM1 at the plasma membrane nor its endocytosis were affected by depletion of flotillins, the authors indicated that flotillins might play a role in endosomal sorting of cargo towards the ER or TGN. In line with these findings, Pust and coworkers analyzed the role of flotillins in the cellular transport of ricin and Shiga toxin [68]. Again, the endocytic uptake of both toxins was not affected by depletion of flotillins, whereas the retrograde transport of the toxins towards the TGN and ER was impaired and caused an accumulation of both toxins, thus increasing their toxicity [68].

Well in line with the above findings, we have recently described a novel role for flotillins in the endosomal sorting of the β-secretase BACE1 [69]. Flotillin-1 binds to a di-leucine sorting motif in the cytoplasmic tail of BACE1 and thereby competes with the adapter protein Golgi-localized, gamma adaptin ear-containing, ADP ribosylation factor binding protein 2 (GGA2) for the binding to BACE1 tail. Previous studies have shown that GGA proteins are important for both the retrograde trafficking and recycling of BACE1 towards Golgi and plasma membrane and for sorting to lysosomes for degradation [25,74,75,76,77,78]. Depletion of flotillins leads to an accumulation of BACE1 in endosomes, which in turn increased the amyloidogenic processing of the Alzheimer amyloid precursor protein (APP) [69]. Our study showed for the first time a direct binding of flotillins to a canonical sorting motif in a transmembrane cargo protein. Based on our data, flotillins might thus participate in cargo sorting towards recycling. This is in line with recent findings by Solis and colleagues, who found that overexpressed flotillins associated with tubulovesicular recycling compartments positive for Rab11a, sorting nexin-4 and EH domain containing-1 in A431 cells [42] and that depletion of flotillins affected the recycling of the transferrin receptor and E-cadherin [42,79]. Thus, a novel and highly intriguing role for flotillins in the regulation of cargo sorting events within endosomes appears to be emerging.

### 4.2. An Indirect Role of Flotillins in Endocytosis: Pre-Endocytic Clustering at the Plasma Membrane

**Figure 1:**
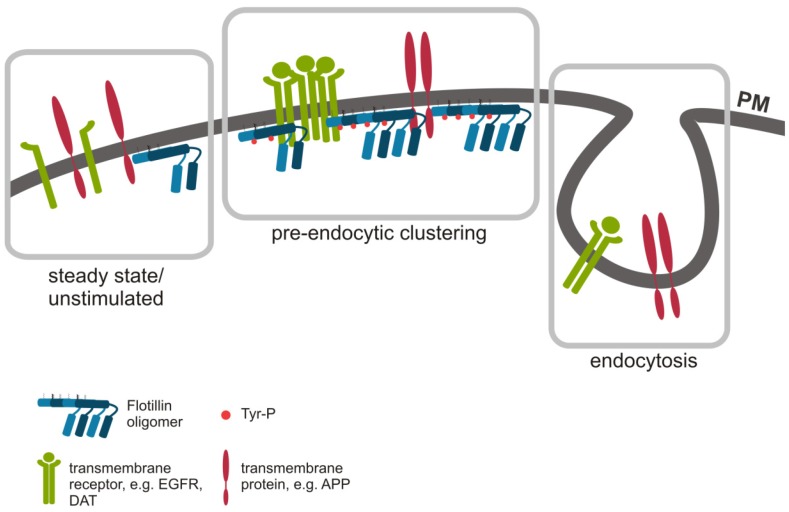
Flotillin assisted endocytosis. Flotillin microdomains are dynamic, and upon certain stimuli, flotillins form higher order oligomers that can recruit transmembrane proteins, such as EGFR, DAT and APP, into flotillin rafts for pre-endocytic cluster formation (middle). Cargo can then be internalized via endocytosis without a direct involvement of flotillins, e.g., by clathrin mediated endocytosis (right). In resting or growth factor deprived cells, flotillins and the cargo may not reside in the same microdomains (left).

Recent findings have casted some doubt on the direct role of flotillins in the endocytic uptake of some cargo molecules, as flotillins were shown to specifically cluster cargo molecules, such as APP, the dopamine transporter (DAT) and the epidermal growth factor receptor (EGFR), at the plasma membrane prior to endocytosis by means of CME [37,57,80]. Figure 1 summarizes the potential role of flotillins in the endocytosis of cargo proteins. Depletion of flotillin-2, but not of flotillin-1, impairs APP endocytosis in neuroblastoma cells and in primary hippocampal neurons [57]. Strikingly, using STED microscopy, Schneider and colleagues showed that APP requires flotillin-2 for the formation of pre-endocytic clusters at the plasma membrane that are necessary for a proper endocytic uptake of APP [57]. Since APP is a classical cargo protein of CME [81,82,83], the authors suggested that APP is internalized by a specialized CME pathway that is regulated by flotillin-2, but the details of the mechanism how flotillins affect CME still await further characterization.

The role of flotillins in the endocytosis of the dopamine transporter DAT has recently been set under debate. While Cremona and co-workers suggested an essential role for flotillins during DAT endocytosis, which goes along with a phosphorylation of flotillin-1 on Ser 315 by protein kinase C (PKC), Sorkina and colleagues established that flotillins actually are necessary for the decreased mobility and clustering of the transporter in the plasma membrane prior to its CME mediated uptake [58,84]. In line with this, we have observed that flotillin-1 influences the clustering of EGFR upon EGF stimulation at the plasma membrane, but not EGFR endocytosis [37]. Even though EGFR has been suggested to utilize both CME or CIE pathways, depending on the ligand dosage [85,86], we did not observe any effect on EGFR uptake upon depletion of flotillin-1 [37], nor did we see a colocalization of flotillins and EGFR in early endocytic vesicles [12,37]. Thus, it appears that although APP, DAT and EGFR are internalized by CME, they all depend on flotillins for preassembly prior to endocytosis. However, a direct molecular connection between flotillin microdomains and clathrin coated structures at the plasma membrane is currently missing, and there is a very limited degree of overlap of clathrin with flotillins at the plasma membrane [20]. Interestingly, there is some evidence suggesting that clathrin coated structures may assemble in plasma membrane microdomains [87], but there are no data as yet if these structures contain flotillins. It is highly unlikely that flotillins function as essential components of CME or coated pit assembly, since endocytosis of clathrin dependent cargo, e.g., transferrin receptor, is not generally impaired upon flotillin depletion (Our unpublished findings). However, it is possible that there may be a specific subset of cargo that is endocytosed by means of CME that takes place from microdomains that contain flotillins.

Interestingly, flotillins contain cholesterol recognition/interaction amino acid consensus (CRAC) motifs and have been suggested to bind cholesterol ([27,88]; our unpublished findings). The transmembrane NPC1L1 protein mediates cellular cholesterol uptake and cycles between the plasma membrane and recycling endosomes [50,89]. Upon its endocytic uptake, NPC1L1 utilizes a CME pathway [89]. Interestingly, by co-immunoprecipitation and FRET analysis, flotillins were shown to associate with NPC1L1 and to be required for a cholesterol induced uptake of NPC1L1 [50]. Furthermore, the presence of NPC1L1 in flotillin microdomains is in line with the binding of flotillins to cholesterol ([50,88]; our unpublished findings) and their scaffolding microdomain activity. Strikingly, Ge *et al.* suggest that flotillins mediate the recruitment of clathrin and its adaptor protein AP-2 to NPC1L1 and thereby facilitate the uptake of NPC1L1 [50,89]. Therefore, flotillins, due to their propensity to form oligomers and to bind cholesterol, might contribute to microdomain scaffolding in the pre-assembly or clustering of cargo proteins destined for endocytosis. Similar observations have been published by Abrami *et al.* [90,91,92] who found that the anthrax toxin is endocytosed by CME, but depends on lipid microdomains for the clustering during CCP assembly [90], as has also been shown for the tetanus neurotoxin [93]. Thus, one could assume a general role for lipid microdomains in the pre-endocytic clustering and assembly of cargo molecules destined for endocytosis, independent of the final endocytosis pathway used.

## 5. Flotillins as Endocytic Cargo during Signaling

So far, most studies addressing flotillins and endocytosis have neglected the possibility that flotillins themselves might be cargo molecules for a CIE pathway. Adding to the controversy as to whether flotillins establish their own endocytosis pathway or only assist in cargo clustering for endocytosis, Glebov and co-workers could not exclude that flotillin-1 might itself be a cargo molecule that is recognized by a CIE pathway [20]. Most studies so far have addressed only steady state endocytic pathways [20,21]. However, it has been conclusively shown that flotillins participate in growth factor signaling and that flotillin microdomains increase in size and translocate to endosomes upon EGF stimulation [12,32,33,37]. These two pathways, the steady state uptake and the growth factor induced translocation, might represent two different routes that flotillins utilize for their internalization. This is also supported by recent findings on dynamin dependency of flotillin uptake. Inhibition of dynamin GTPase activity showed that EGF mediated flotillin uptake requires dynamin activity [22], whereas uptake of some of the suggested flotillin cargo molecules appears to be dynamin independent (reviewed in [23]). However, flotillin uptake – irrespective of whether as a cargo molecule or as a structural endocytic component – depends on cholesterol [50,57]. Dissection of the role of dynamin in the cellular trafficking of flotillins is complicated by the fact that expression of dominant-negative dynamin mutants (K44A, T65A or R399A) impairs the recycling of flotillins from endosomes to the plasma membrane, which also, somewhat surprisingly, appears to require clathrin [22]. However, both dynamin and clathrin have been shown to participate in sorting events in endosomes that mediate recycling of cargo towards the plasma membrane [94,95], making their role in the recycling of flotillins plausible.

## 6. A New Era: From Flotillin Dependent to Flotillin Assisted Endocytosis

Due to the findings showing that flotillin depletion reduces the uptake of some proteins from the plasma membrane, the term “flotillin dependent endocytosis” has been established. However, this term implies that flotillins are essential mechanistic components of a specific endocytic pathway that is at least severely impaired in their absence (as with “clathrin dependent”). In the case of clathrin and dynamin, the dependency is well established, and the use of the word “dependent” well justified. However, as the evidence for a similar, essential mechanistic role for flotillins and the details of the nature of the endocytic carriers are currently lacking, we suggest that the term “flotillin assisted endocytosis” should rather be used. In our opinion, the word “assisted” describes a process that is facilitated by flotillins (e.g., by cargo sequestering prior to endocytosis) but is not strictly and mechanistically dependent on flotillins as structural component. Thus, as long as the essential nature of flotillins in the endocytosis of a specific set of cargo that is endocytosed by means of the said pathway has not been shown, we feel that “flotillin assisted endocytosis” is currently more adequate. 

## 7. Conclusions: Flotillins in Membrane Trafficking–Getting the Bigger Picture

**Figure 2:**
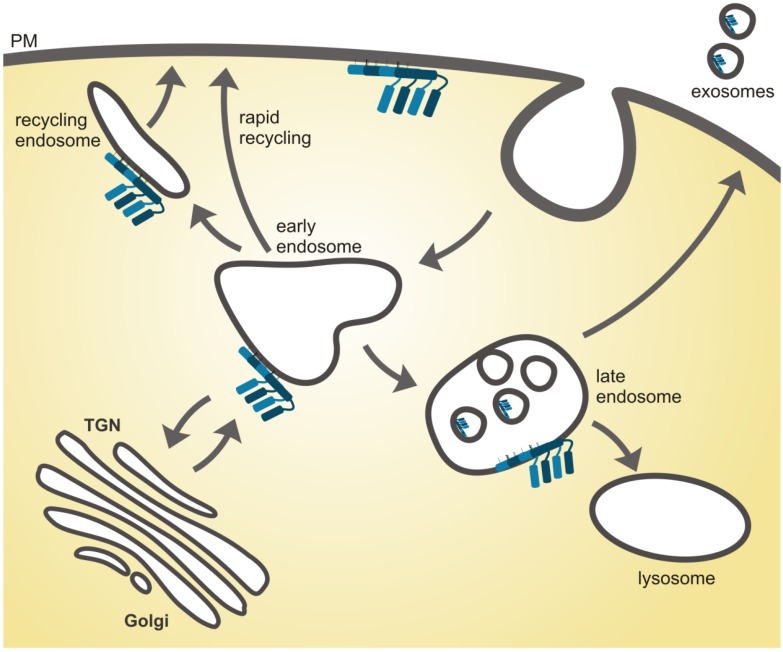
Function of flotillins in cellular cargo sorting processes. Flotillin microdomains have been described at the plasma membrane, in early, late and recycling endosomes as well as in exosomes. At the plasma membrane, flotillins assist transmembrane cargo proteins during cluster formation prior to endocytosis. In endosomes, flotillins appear to be involved in cargo sorting towards recycling to the plasma membrane, retrograde transport to the Golgi and ER or to intraluminal vesicles of multivesicular bodies, which then fuse with the plasma membrane and release the internal vesicles as exosomes.

The above findings show that although flotillins undoubtedly regulate various membrane trafficking events of numerous cargo proteins, the exact step that involves flotillins needs to be carefully dissected to avoid wrong conclusions. In Figure 2, we have summarized the various trafficking steps in which flotillins have been suggested to play a functional role. To define and substantiate the molecular details of an endocytic pathway that depends on flotillins, further evidence needs to be gathered. For example, several studies showed that flotillins colocalize with their putative cargo, e.g., CD59, in early endosomes. Strikingly, most cargo molecules, irrespective of their internalization route, merge in early or sorting endosomes, and therefore, a colocalization in early endosomes does not provide a final proof that the two proteins arrived via the same endocytic uptake route. To visualize that two specific proteins take the same internalization pathway, photo-activatable tags in combination with live imaging should be used. On the other hand, such analysis is always based on ectopic expression of tagged proteins, which may not be identical with the endogenous ones. As already indicated by Glebov *et al.*, isolation of flotillin positive endocytic vesicles with a consecutive mass spectrometric analysis would help characterize the nature of these vesicles and facilitate the identification of structural as well as accessory proteins. The isolation of flotillin positive vesicles could be done in two ways, in order to distinguish between flotillin assisted endocytosis and endocytosis of flotillins as cargo: (1) As described by Glebov *et al.* using the fluid phase uptake of ferrofluid to analyze vesicles generated by steady-state uptake; and (2) using ferrofluid coupled to EGF to analyze the growth factor induced translocation of flotillins and the respective carrier vesicle composition. To address the question whether flotillins serve as structural components of endocytosis, it would be necessary to not only observe flotillin containing regions with electron microscopy but also to understand the protein structure of flotillins and to define how flotillins might induce invaginations and buds at the plasma membrane.

In the future, another important issue will be to dissect the details of flotillin function in endosomal sorting. This will require the identification of cargo molecules—proteins as well as lipids and toxins—that are sorted within the endosomal system by means of flotillin microdomains. Some of the potential cargos have been identified by us and others [67,68,69], but the next challenge will be to identify the accessory proteins and adaptors involved in flotillin mediated sorting. So far, none have been characterized for flotillin assisted endocytic pathways, whereas our findings strongly suggest that the adaptors of the GGA family might be involved in endosomal cargo sorting by flotillins [69]. It will also be important to dissect how flotillins interact with the cargo and the accessory proteins/coats during their sorting function.
